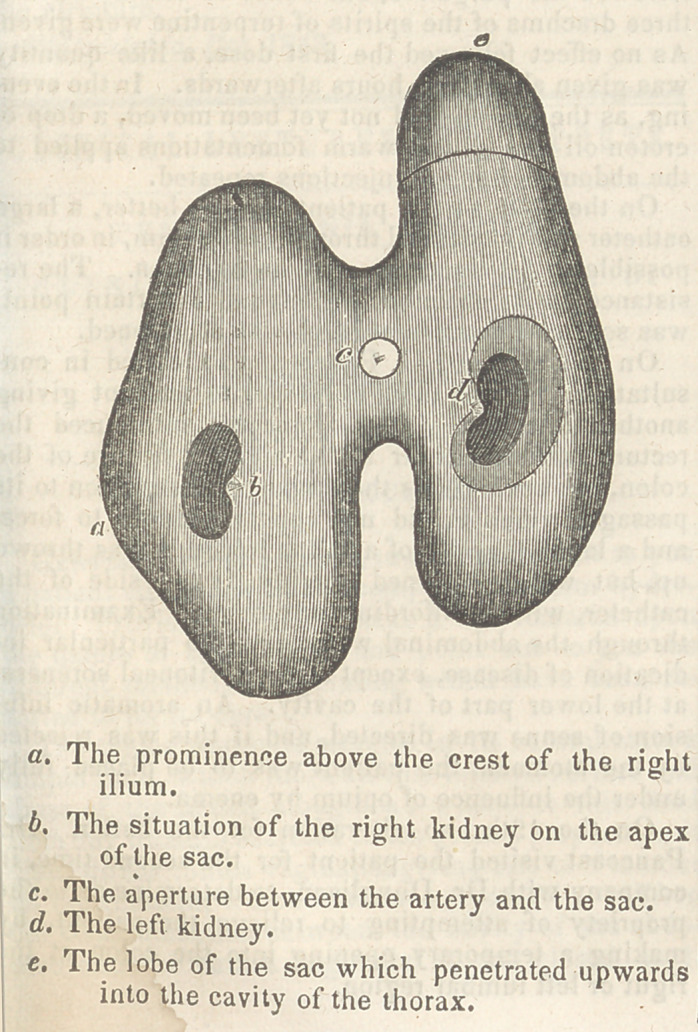# A Case of Dissecting Aneurism of the Aorta

**Published:** 1843-12-09

**Authors:** E. Michener

**Affiliations:** New Garden, Chester Co., Pa.


THE MEDICAL EXAMINER,

And Retrospect of the Medical Sciences.

Vol. VI.] PHILADELPHIA, SATURDAY, DECEMBER 9, 1843. [No. 24.

### A CASE OF DISSECTING ANEURISM OF THE AORTA.

BY E. MICHENER, M. D.

10th mo. 14th, 1843.—Nelson Taylor, aged 33 years, was attacked more than a year ago with pain in the lumbar region, which after some days left him, but continued to recur at intervals for some months. For this he was variously treated by several physicians of Philadelphia,—sometimes for a spinal, sometimes for a renal affection. His health still declining, he came to the country in the Sixth month last, where he became the patient of Dr. Boyd, who likewise considered his case one of kidney disease, and treated him accordingly.

About six weeks ago, when lying perfectly still in bed, on his back, he experienced a sensation in the right iliac region, which he compared to something of the size of the finger being thrust suddenly forward into the flesh, and in an instant after the same sensation was repeated. This was immediately followed by acute pain, which extended down the limb, and rendered it almost useless for two or three days. Pains of a similar character have occurred in the lower extremities—but mostly in the *left* one—at intervals ever since. Three weeks afterwards Dr. Boyd discovered a circumscribed tumour, two or three inches in diameter, lying low down in the right iliac region. The tumour was not tender on pressure, nor did he observe any pulsation in it. From that time forward it rapidly extended upwards and forwards, displacing the viscera to the left side. Three days ago Dr. Boyd detected pulsation in the tumour. The strength of the patient has rapidly declined during the last six weeks.

Last evening I received a note from Dr. Boyd, expressive of the mutual wish of Nelson and himself that I should see the case this morning. He did not attend. I found a tense, elastic tumour, occupying the space between the umbilicus and crest of the right ilium, of a spherical form, and not less than six inches in diameter. It is not painful, neither is it tender on pressure; but there is a strong, *undulatory*, pulsating motion, synchronous with that of the heart, and so strong as to be seen at several yards distance, when uncovered. The circulation in the limbs below does not appear to be affected. He has slight symptoms, resembling hectic, but these may depend upon the use of morphine, which he takes for the paroxysms of pain in the left lower extremity.

Notwithstanding the rapid progress of the tumour, the want of pulsation in its commencement, and the undulating character of its motions, I gave it as my opinion that it was an aneurism, and probably proceeded from the common iliac. I advised only a palliative treatment.

The tumour is gradually increasing in all directions, and the pulsation is particularly strong in the side above the crest of the ilium.

19th. Dr. Seal saw the patient with me, and fully concurred in my opinion.

The tumour now presents a distinct lobe just below the margin of the ribs, with a strong sulcus between. The original tumour still increases, and the pulsation grows stronger. Paroxysms of pain in the left extremity are frequent and severe, requiring large doses of morphine. He takes little nourishment; has a general exsanguineous appearance, and is fast declining in strength.

21st. The tumour now extends from the pubes to the margin of the ribs; is most prominent just within the ilium; and the sinus mentioned in last report is nearly filled up. He is very feeble, and takes nothing but cold water.

Advised him to take light nourishing food.

24th. Met Drs. Boyd and Pennock this morning. The latter concurred with me.

The tumour still progresses, and the whole abdomen seems quite too full. The pulsations are so strong, that when the stethoscope is employed the head of the operator is raised nearly a quarter of an inch by every impulse of the heart. Were it not for this positive indication, it would seem to me almost incredible that an aneurismal sac could reach such an enormous size without rupturing. Nor does the extraordinary progress of the present case accord well with that of aneurisms in general. Both these difficulties may, however, be partially solved by the observations of Morgagni and other surgeons, that in large aneurisms of the abdominal aorta the sac is only in part made up of the coats of the vessel, constituting mixed aneurisms.

In the afternoon, Drs. Worthington and Thomas saw the patient, in company with Dr. Boyd. They fully admitted the obscurity of the diagnosis, but hesitatingly agreed with Dr. Boyd that it was an abscess. They were led to this conclusion by the early history of the case, by the want of pulsation at the commencement of the tumour, and by its very rapid developement. But the circumscribed form of the tumour from the first, its remarkable pulsation, and the entire absence of irritative fever, still inclines me to adhere to my first opinion. Besides, while the tumour was small, and lay deeply imbedded among the abdominal viscera, the pulsation might have been overlooked by Dr. Boyd.

28th. A note was received to-day from Drs. Worthington and Thomas, stating that on further reflection, since their return home, they had reversed their former opinion, and now concurred with those who considered the case to be aneurism. They also informed me that they intend visiting him to-morrow, in company with Professor Gibson, of Philadelphia.

29th. Met Drs. Gibson, Worthington, Thomas, Pennock and Boyd. After a full examination, Professor Gibson pronounced the case to be one of aneurism; premising, however, that the symptoms were unusually obscure, and that this might be occasioned by some undiscoverable complication of disease. He supposed that it might have originated from the aorta near its bifurcation. All present seemed to unite in the general accuracy of these views, and concurred in the continuance of only a palliative treatment.

The pulsation is sensibly weaker, but the tumour is still increasing. The strength continues to decline; and there is increased sallowness of the skin.

11th mo. 2d. For the last two days he has taken more food; has suffered less from paroxysms of pain; and in other respects has felt more comfortable, though constantly declining in strength.

Advised the moderate use of wine.

6th. The tumour has constantly increased, especially in the direction downwards, and in front of the ilium. The pulsation is greatly reduced in strength. He feels invigorated by the wine; suffers less from pain; takes a larger portion of food, but is more disposed to wakefulness; and his pulse, voice, and general condition all indicate a rapid reduction of the vital powers, or rather, of the available quantity of vital fluid in the system. In consequence of a slough on the sacrum, he has laid much of the last two days on the right side.

10th. Has slept but little for several nights. During the past night he experienced a sensation which he supposed was caused by a rupture of the sac, and induced him to have the family called up. Suffered a severe paroxysm of his usual pain this morning, which left him completely exhausted. Several spots of diffuse gangrene appear on and around the sacrum. Takes no food; extremities cool; pulse very frequent and feeble.

For the last two days the tumour has increased rapidly in the direction of the scrobiculus cordis. The abdomen is very full, and tense; and the epigastrium is painful on the slightest touch. The swelling causes considerable oppression, and renders respiration laborious. These concurrent signs of a speedy crisis led me to apprize the family of his approaching dissolution.

11th. I was called to see him at 9 o'clock last evening, in consequence of acute pain in the epigastrium. I found him in a state of severe suffering; epigastrium very tense, and intolerant to pressure even of light clothing; pulse 160, weak; respiration quite laborious; extremities cold, but the intellect clear. The pain abated on taking some morphine, but the oppression increased till midnight, when he seemed to lose his consciousness. From this time he talked incoherently, and continually passed his hands over the abdomen, indicating a state of suffering there, until three o'clock, when he became perfectly still, and at half past four, A. M., expired without a struggle.

Immediately after death I observed that the tumour was neither so tense nor prominent as before, yet I had no reason to suppose that the sac had given way.

Agreeable to his own request, arrangements were made for a *post-mortem* examination.

12th. We proceeded to the examination, thirty-four hours after death. Present, Doctors Worthington, Pennock, Hobson, Bye, D. M. Alison, Boyd and myself. This late period was chosen in order to accommodate those who live at a distance, without adverting to the tendency which such a mass of blood must have to run into rapid putrefaction.

On exposing the body, the parts covering the tumour—indeed, nearly the whole of the abdominal parietes were almost black, and quite offensive. From this and other causes, which will become manifest as we proceed, both the operation itself and the discrimination of parts were rendered difficult. I shall follow the course of the dissection, which was hastily made, as closely as possible.

The abdomen was laid open by a double crucial incision. The peritoneum contained a small quantity of bloody serum; and the intestines were all crowded into the left side, except the cœcum, which was strongly adherent to the apex of the tumour. The omentum and intestines, in the immediate vicinity of the tumour, had formed slight adhesions, which were easily separated with the fingers. The stomach and intestines, together with the omentum and mesentery, were removed. The tumour, which now presented, occupied the entire right side of the cavity, and, with the whole posterior aspect of the abdomen, exhibited one continuous mass of black grumous blood, with which the sub-peritoneal cellular tissue was completely infiltrated. No trace of the right kidney was to be seen. The left one was thrust forward, and appeared of two or three times its ordinary size. The artery was now traced from above the bifurcation of the aorta along the common, internal, and external iliacs, all which were in a normal state. Dr. Boyd next attempted to detach the sac along its spinal margin, but penetrated immediately into the cavity throughout its whole extent.

The sac was then everted over the ilium, and a very large wash bowl completely filled with its contents, consisting of concentric laminæ of half organized lymph, coagula, and fluid blood. The sac was composed of the peritoneum and subjacent cellular tissue, so completely infiltrated with blood as to be distinguished with the greatest difficulty from the parts within. It was impossible to say exactly where the one terminated or the other commenced. Posteriorly the sac was formed of the psoas, the iliacus, and the abdominal muscles. Thus far we had not discovered a vestage of the right kidney, but it was now found imbedded in the ecchymosed cellular tissue, which formed the anterior portion of the sac. It appeared to be in a healthy condition, although it must have been protruded forwards more than six inches, and lay in front of, and just above the superior anterior spinous process of the ilium. The liver and spleen, with large portions of the sac and great quantities of blood were next removed, in order to trace the aorta upwards. In doing this we discovered a diseased state of the spinal column. The two lower dorsal and two upper lumbar vertebræ were denuded of periosteum on their anterior aspect, and the bodies of the two intermediate ones were much wasted. On raising the artery, an aperture was brought into view, in its posterior side, large enough to admit the thumb. It was nearly circular; the edges were smooth, rounded, and resembled an old cicatrix. The vessel was slightly dilated, and bore marks of former disease at the point of perforation; and it was completely consolidated with the sac all round the orifice. The aperture was a little inclined to the right side of the spine, and lay opposite to the articulation of the last dorsal and first lumbar bones.

After having removed the diseased vertebræ, and cleansed them from adherent blood, I found the bodies, and much of the anterior aspect of the lateral processes, denuded of their periosteum, and apparently in a state of *necrosis;* for the osseous matter being dry, firm, and free from the well known odour and secretion accompanying carious bone. The whole of the denuded surfaces were neatly covered with a smooth pellucid coat of firm lymph, of the thickness of paper, which could be peeled off, leaving the bone quite clean. At the upper edge of the first vertebræ, and a little to the right of the median line, corresponding exactly with the aperture in the aorta, is a salient projection of bone. It is probable that the vessel was perforated by the more or less direct action of this point. It may also be proper to remark, that the deepest excavations in the bones were immediately opposite the aperture in the artery, where the jet of blood would impinge with the greatest force. Pursuing our investigations across the spine into the left side, we entered another lobe of the sac, which, like the one on the right, extended downwards behind the left kidney, elevating it two or three inches, and finally reaching to the pelvis. On this side the blood also took a direction upwards, burrowing behind the spleen, tearing up the posterior attachments of the diaphragm, and raising the pleura for four or five inches within the cavity of the thorax. The hypertrophied appearance of the left kidney, which has been noticed, was only *apparent*, and was produced by the infiltration of blood into the cellular capsule, by which it is surrounded. Indeed, so far as they were examined, we discovered no appearance of organic lesion in any of the viscera.

There were no actual measurements taken, either of the dimensions or of the capacity of this enormous sac; but if we take the right and left lobes together, they must have exceeded twenty-four inches in length, and at its most prominent part, opposite the crest of the ilium, its antero-posterior diameter could not have been less than eight inches. The most accurate estimate of its contents which I can arrive at is derived from the bowl, which was filled with them. It must have contained at least *six quarts;* and if all the remaining portion had been collected, it would certainly have measured as many more—thus giving *three gallons* as the contents of the tumour.

The accompanying diagram will afford a tolerable idea of the outline of the sac.

*a.* The prominence above the crest of the right ilium.

*b.* The situation of the right kidney on the apex of the sac.

*c.* The aperture between the artery and the sac.

*d.* The left kidney.

*e.* The lobe of the sac which penetrated upwards into the cavity of the thorax.

I am aware that this statement will impose a severe tax upon the credibility of the narrator among those who did not see for themselves; for it is difficult to conceive how the exhausted powers of the system could have endured so long, and to so great an extent, the drainage of the vital fluid from its proper receptacles. But it has been my endeavour to give a faithful, though very imperfect narration of the case.

*Remarks.*

It may be instructive to review some of the circumstances which produced so much obscurity in the foregoing case, and endeavour, by the light of dissection, to trace the phenomena which it presented to their proper causes.

All who saw the case acknowledged the obscurity of the diagnosis. At times the opinions entertained were entirely irreconcilable; and it must be admitted that, until dissection revealed its true character, the precise nature of the lesion was not understood by any. The general opinion prevailed that it was aneurism, but no one could define its characters. All must now agree that it was a *false* or *cellular aneurism.* The absence of adhesion between the laminæ of the tissue composing the sac allowed of its very rapid expansion, and permitted even the sac itself to become completely infiltrated with the contained blood.

It seems probable that the spine first became affected, so as to produce the salient point of bone which I have described. The vessel was then abraded, so as to induce ulceration, or more directly cut through. At first the aperture would necessarily be small, and the blood would escape slowly. This would allow time for the formation of a cellular sac, capable of resisting the constantly increasing jet of blood, to a degree at least sufficient to prevent the otherwise immediate consequences of opening so large an artery. The jet of blood having to impinge directly against the spine would lose much of its force, and this circumstance would very much aid the resistance of the surrounding tissues. These views are confirmed by the facts, that the projecting bone is a little to the right of the median line, the aperture in the vessel corresponded exactly with it, and the greater mass of blood had taken that direction.

However satisfactory the *rationale* just offered may appear, I cannot refrain from suggesting another for consideration. If we admit the ulceration of the coats of the artery as the primary lesion, it seems not unreasonable to suppose, that the incessant force of the jet of blood impinging against the spine might cause the absorption of the periosteum and subjacent bone, so as to produce the condition described. In this case, the deeper perforations, which extended nearly through to the spinal canal, and which were found opposite to the orifice in the artery, may have been worn away by the impulsive force of the blood after the bones had lost their vitality.

At first the blood probably took a direction downwards behind the right kidney, by which it was strongly tied down. Having passed below that viscus it found less resistance, and pushed forwards into the iliac region, where it formed the prominent tumour discovered by Dr. Boyd. This view explains the absence (or perhaps I should say the feebleness) of pulsation in the tumour at an early period. The jet was first thrown against the spinal column, as we have already seen, and the reflex current, with a greatly subdued force, had then to traverse a narrow confined passage behind the kidney before it reached the tumour in the iliac region. The position of the cœcum on the very apex of the sac, would add still more to the obscurity.

The sulcus, which was at one time very apparent on the anterior aspect of the tumour, was occasioned by the kidney, which probably did not part from its attachments quite so readily as the surrounding tissues. This position of the kidney enables us to understand why the pulsation was always less distinct at that point than in the side above the ilium.

The most remarkable and characteristic symptom was what I have termed the "undulatory pulsation" of the sac, in contradistinction from the bold and forcible stroke observed in true aneurisms of the aorta. I will leave it for others, who possess better opportunities, to decide the question; but it appears to me that the difference in the texture, and, consequently, in the resistance offered by the two kinds of sac, must always produce a corresponding difference in the character of its pulsations. If further observations shall confirm this opinion, the character of the pulsation will afford a means of discriminating between the *true* and *false* aneurism.

It has been stated that the pulsations in the aneurism were synchronous with those of the heart. This was at one time doubted by some who made the examination. It may be proper to offer an explanation of the source of this error. While the pulse remained slow and full there was no difficulty, but as his strength declined, and the pulse grew more frequent and feeble, it became more uncertain. It necessarily required a longer interval of time for the slow undulations to pass through the sac than what the accelerated pulsations of the heart allowed. Each *succeeding* undulation commenced before the *preceding* one had ceased—rendering them confused and indistinct. In the latter period of the disease, the synchronism of the undulations could be determined much more satisfactorily by the sight than by the touch.

I have said nothing of the stethoscopic characters of the case, because, to my unpractised ear, its oracular revelations too often afford an uncertain sound. But auscultation was not neglected.

In all its stages, after the pulsation was discovered, the tumour yielded that *rushing sound* which is considered to be pathognomonic of aneurism. The *whizzing sound*, produced by the blood escaping from a fissured vessel, could not be so clearly ascertained; yet Dr. Seal believed, on more than one occasion, that he could detect it *high up* in the epigastrium.

Dr. Pennock and myself both succeeded in tracing the pulsation of the artery, distinct from that of the tumour, along the aorta, the common, and the external iliac arteries, from whence we inferred that there might be a *fissure* of the vessel, without so far destroying its integrity as to prevent the free passage of blood through it. This view was confirmed by the uninterrupted circulation in the parts below the aneurism.

In all these particulars, the diagnosis afforded by the stethoscope was found to be correct.

*New Garden, Chester Co., Pa.*, 11th mo. 15, 1843.

---

HOW TO MAKE LEECHES BITE.—The leech, which it is intended to apply, is to be thrown into a saucer containing fresh beer, and is to be left there till it begins to be quite lively. When it has moved about in the vessel for a few moments, it is to be quickly taken out and applied.—*Med. Gaz., from Weit. Bietr.*

## Figures and Tables

**Figure f1:**